# “Uterine Dehiscence: A Diagnostic Conundrum in Repeat Cesarean Deliveries”

**DOI:** 10.1002/ccr3.70791

**Published:** 2025-08-13

**Authors:** Pamela Sarue, Nicholas Eynon, Reine A. Zbeidy

**Affiliations:** ^1^ Division of Obstetric Anesthesiology University of Miami, Jackson Memorial Hospital Miami Florida USA; ^2^ University of Miami Miller School of Medicine Miami Florida USA

**Keywords:** anesthesia, fetal medicine, obstetrics/gynecology, perinatal medicine

## Abstract

Uterine dehiscence (UD), often asymptomatic and underdiagnosed, is a significant risk in patients with prior cesarean deliveries. Serious complications include uterine rupture; increasing maternal and neonatal morbidity. Improved diagnostic protocols, particularly antenatal imaging, are essential to differentiate UD from placenta accreta spectrum, optimize resource utilization, and enhance patient outcomes.

## Graphical abstract

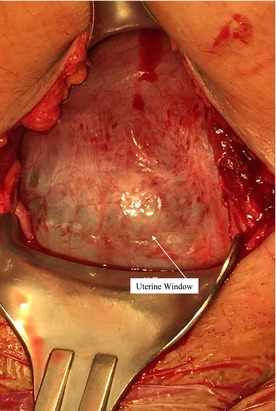

A 31‐year‐old G5P4004 woman was scheduled for repeat cesarean delivery (CD). She had four prior CDs, anterior placenta, and suspected placental accreta, which was intraoperatively diagnosed as uterine dehiscence (UD) (Figure 1). Most UD cases are asymptomatic with no bleeding [1], and the lack of standard diagnostic protocol makes their evaluation and management challenging [1].

**FIGURE 1:**
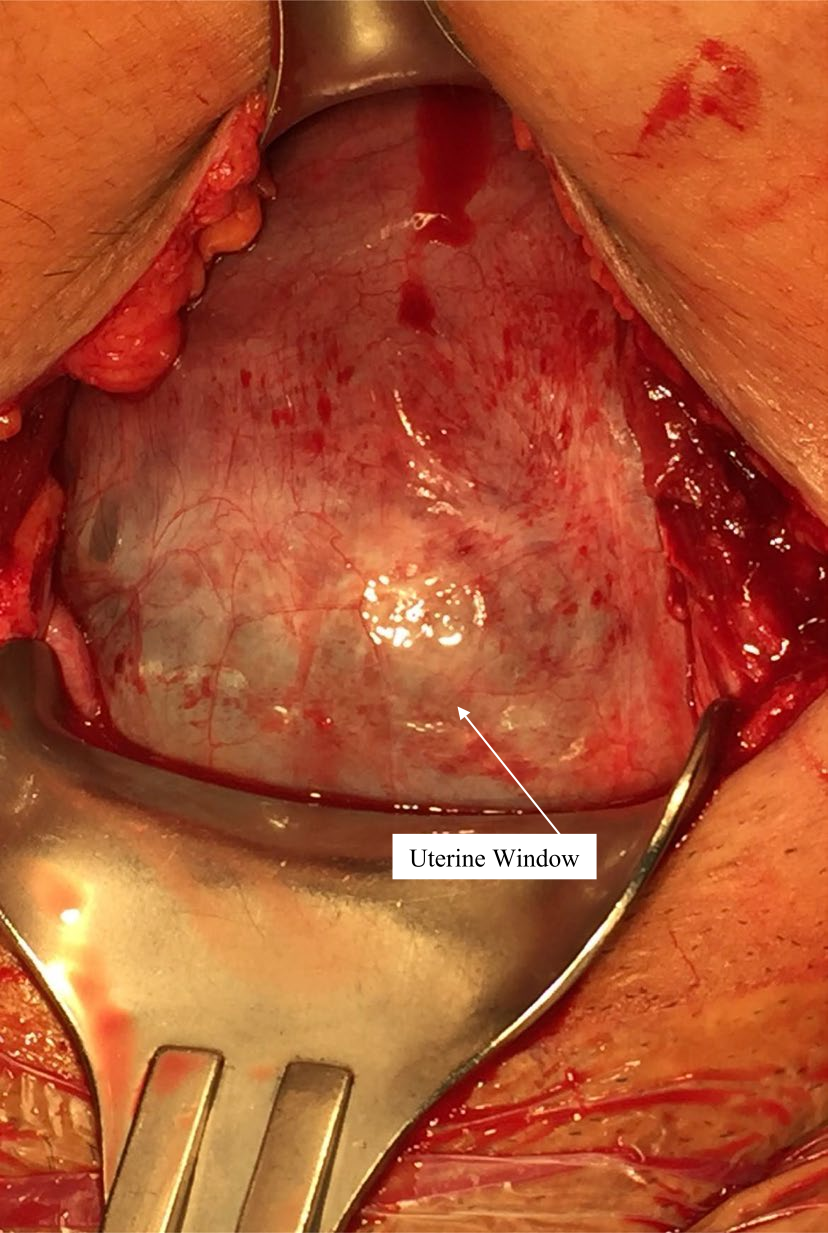
Intraoperative photograph demonstrating a markedly thinned and bulging lower uterine segment at the site of the old cesarean scar, consistent with uterine dehiscence.

UD is a partial division of the uterus that does not affect all three layers (endometrium, myometrium, and perimetrium) [1]. It is more common with each additional prior CD [1]. With CD rates increasing from 5% to 30% in the past 35 years [1], this condition merits attention.

UD may lead to a “uterine window,” a thin uterine wall segment that reveals the fetus through the myometrium, as illustrated in Figure 1 [1]. Often undiagnosed due to the lack of a diagnostic protocol, it can be identified intraoperatively during repeat CD or antenatally between pregnancies on transvaginal ultrasound [1]. However, there remains significant variability in the reliability and interpretation of antenatal imaging findings.

Transvaginal ultrasound with lower uterine segment (LUS) thickness measurement is a noninvasive method increasingly used to assess scar integrity in patients with prior CD. LUS thickness < 2.5 mm has been associated with increased risk of uterine rupture and UD [2]. Yet, no universally accepted cutoff exists, and variability in technique and patient factors (e.g., gestational age, fetal position) can limit predictive value [2]. Color Doppler imaging, while more often utilized in the evaluation of placental accreta spectrum (PAS), may offer additional diagnostic clues when differentiating between PAS and UD. PAS is typically associated with placental lacunae, loss of the hypoechoic retroplacental zone, and turbulent blood flow with bridging vessels extending beyond the uterine serosa—features usually absent in UD [2].

Importantly, the preoperative misdiagnosis of a uterine window as placenta accreta spectrum demands additional resources such as more invasive monitoring, blood product availability, and multiservice involvement, all of which could be avoided with more accurate diagnostic tools. This underscores the necessity for advanced diagnostic modalities for UD and windows [2].

A retrospective study of 21,420 patients with a history of CD reported UD in 10.1% and uterine rupture—the most concerning complication of UD—in 2.8% of cases [3]. These findings reinforce the importance of enhanced antenatal risk stratification, given that uterine rupture is associated with substantial maternal and neonatal morbidity [1]. The potential for rapid hemodynamic deterioration reinforces the importance of maintaining clinical vigilance in patients with multiple prior cesarean deliveries and atypical imaging findings.

This case reinforces the need for heightened clinical suspicion of UD in high‐risk patients and highlights the need for more refined, evidence‐based standardized imaging criteria to more accurately differentiate UD from PAS in the peripartum setting.

## Author Contributions

**Pamela Sarue:** conceptualization, investigation, project administration, writing – original draft, writing – review and editing. **Nicholas Eynon:** writing – review and editing. **Reine A. Zbeidy:** conceptualization, investigation, project administration, supervision, writing – original draft.

## Disclosure

Clinical Trial Number and Registry: Not applicable.

Prior Presentations: Not applicable.

Abbreviated Title: Not applicable.

Summary Statement: Not applicable.

## Consent

Patient consent signed and collected in accordance with the journal's patient consent policy.

## Conflicts of Interest

The authors declare no conflicts of interest.

## Data Availability

Data sharing not applicable to this article as no datasets were generated or analysed during the current study.
